# Human naïve regulatory T-cells feature high steady-state turnover and are maintained by IL-7

**DOI:** 10.18632/oncotarget.7512

**Published:** 2016-02-19

**Authors:** Susana L. Silva, Adriana S. Albuquerque, Ana Serra-Caetano, Russell B. Foxall, Ana R. Pires, Paula Matoso, Susana M. Fernandes, João Ferreira, Rémi Cheynier, Rui M. M. Victorino, Iris Caramalho, João T. Barata, Ana E. Sousa

**Affiliations:** ^1^ Instituto de Medicina Molecular, Faculdade de Medicina, Universidade de Lisboa, Lisboa, Portugal; ^2^ Clínica Universitária de Imunoalergologia, Hospital de Santa Maria, Centro Hospitalar Lisboa Norte, Lisboa, Portugal; ^3^ Clínica Universitária de Medicina II, Hospital de Santa Maria, Centro Hospitalar Lisboa Norte, Lisboa, Portugal; ^4^ Infection, Immunity and Inflammation Department, Institut Cochin, INSERM U1016-CNRS UMR8104-Université Paris Descartes, Paris, France

**Keywords:** human regulatory T-cells, naïve regulatory T-cells, regulatory T-cell homeostasis, thymectomy, IL-7, Immunology and Microbiology Section, Immune response, Immunity

## Abstract

Naïve FoxP3-expressing regulatory T-cells (Tregs) are essential to control immune responses via continuous replenishment of the activated-Treg pool with thymus-committed suppressor cells. The mechanisms underlying naïve-Treg maintenance throughout life in face of the age-associated thymic involution remain unclear. We found that in adults thymectomized early in infancy the naïve-Treg pool is remarkably well preserved, in contrast to conventional naïve CD4 T-cells. Naïve-Tregs featured high levels of cycling and pro-survival markers, even in healthy individuals, and contrasted with other circulating naïve/memory CD4 T-cell subsets in terms of their strong γc-cytokine-dependent signaling, particularly in response to IL-7. Accordingly, *ex-vivo* stimulation of naïve-Tregs with IL-7 induced robust cytokine-dependent signaling, Bcl-2 expression, and phosphatidylinositol 3-kinase (PI3K)-dependent proliferation, whilst preserving naïve phenotype and suppressive capacity. Altogether, our data strongly implicate IL-7 in the thymus-independent long-term survival of functional naïve-Tregs, and highlight the potential of targeting the IL-7 pathway to modulate Tregs in different clinical settings.

## INTRODUCTION

FoxP3-expressing regulatory T-cells (Treg) maintain tolerance to self and to the environment, and are central players in the control of immune responses in general [1]. Notwithstanding their relevance in limiting immune-mediated pathology, and their therapeutic potential, research focused on human Treg homeostasis has been scarce [2]. Human Tregs comprise a compartment of thymus-generated naïve-like cells (naïve-Tregs) that continuously replenish the pool of fully-suppressive activated Tregs expressing memory markers [1-7]. The mechanisms governing naïve-Treg homeostasis, in parallel with age-associated thymic involution, remain largely unknown. Moreover, the investigation of naïve-Treg homeostasis has been hampered by the difficulty in clearly identifying this subset in murine models [8].

The establishment and maintenance of the human naïve T-cell compartment is known to rely on both thymic output and peripheral expansion, with their relative contributions still the subject of intense debate. Increasing evidence points to a major contribution of peripheral proliferation in order to explain the relatively stable size of the naïve compartment in adulthood despite thymic involution [9, 10].

Individuals submitted to total thymectomy in early infancy due to corrective cardiac surgery provide an ideal clinical setting to address the peripheral contribution to long-term maintenance of naïve T-cell subsets. Contraction of the entire naïve compartment upon thymectomy has been consistently reported, confirming the central role of the thymus [11-14]. Notwithstanding this, distinct homeostasis of the naïve-Treg and conventional naïve (naïve-Tconv) CD4 T-cell compartments have been suggested by a longitudinal study that showed naïve-Treg preservation up to 1 year post-thymectomy [15], although long-term data are lacking.

Peripheral homeostasis of naïve-Tconvs relies upon a slow rate of cell-turnover resulting from both cytokine-driven proliferation, and TCR-stimulation by low-affinity self-peptides [16]. Additionally, naïve-Tconv homeostasis is known to rely on pro-survival factors, such as Bcl-2, which are up-regulated by homeostatic cytokines, particularly IL-7 [16, 17].

Naïve-Tregs are characterized by high-affinity for self-peptides, and by rapidly differentiating into memory-Tregs upon T-cell receptor (TCR)-stimulation [1-6]. Thus, major histocompatibility (MHC)-self peptide stimulation is likely to result in loss of their naïve phenotype. On the other hand, in terms of the contribution of cytokine-driven proliferation, naïve-Tregs express low levels of receptors for the main homeostatic cytokines. CD25, the α-chain of the IL-2 receptor, is expressed at only intermediate/dim levels [1], questioning whether naïve-Tregs, like their memory counterparts, depend on IL-2 [2]. Moreover, Tregs *per se* typically express low levels of the α-chain of the IL-7 receptor (IL-7Rα), and there are controversial reports on the IL-7 impact on human and murine Tregs [18-22].

We investigated here the impact of IL-7 and IL-2 on peripheral naïve-Tregs from blood and secondary lymphoid organs (SLO), as well as on mature FoxP3^+^ thymocytes, and provide evidence for a role of IL-7 in human naïve-Treg homeostasis. We show for the first time that naïve-Tregs feature much higher levels of the pro-survival molecule Bcl-2 and significantly higher turnover than naïve-Tconvs in healthy individuals. These parameters further increased in the absence of thymic replenishment, ensuring the long-term maintenance of the naïve-Treg compartment in total thymectomized individuals.

## RESULTS

### Preservation of the naïve-Treg compartment following thymus removal

Adults submitted to total thymectomy early in life provide a unique setting to investigate human naïve compartment homeostasis. However, published studies have been hampered by the lack of clear information regarding possible residual thymic activity that can result from either ectopic thymus or post-thymectomy regeneration [11, 12, 14, 23]. We applied here strict criteria to exclude residual thymic activity based on detailed surgical reports and single-joint TCR excision circles (sjTREC) levels clearly below the lowest level observed in healthy adults (Table 1). sjTRECs are by-products of TCR rearrangements during T-cell development that are progressively lost as cells divide in the periphery, and thus used to identify recent thymic emigrant cells [24]. Adults, with a median of 21 years (18-24.5) after total thymectomy, were compared with age-matched healthy individuals (Table 1).

**Table 1:** Characterization of the cohorts

	Healthy	Thymectomized
Number	22	7
Age, years	21 [18-30]	24 [22-27]
Thymectomy, month of age	NA	23 [12-72]
sjTRECs/μl	16.6 [4.1-39.3]	0.5 [0.1-1.8]a
Lymphocytes/μl	2413 [1430-3502]	2010 [933-2618]
% CD4 T-cells	40 [29-52]	43 [23-47]
% Naïve^b^ in CD4	40 [29-58]	15 [9-49]^a^
% FoxP3^+^ in CD4	2.9 [1.2-5.4]	4.1 [3.0-5.6]
FoxP3^+^ cells/μl	21 [9-51]	28 [11-39]
Serum IL-7, pg/ml	15 [7-23]	14 [8-20]

Data are presented as median with limits in square brackets. NA - not applicable.

a P<0.0001.

b Naïve cells defined as CCR7+CD45RO─­

**Figure 1:**
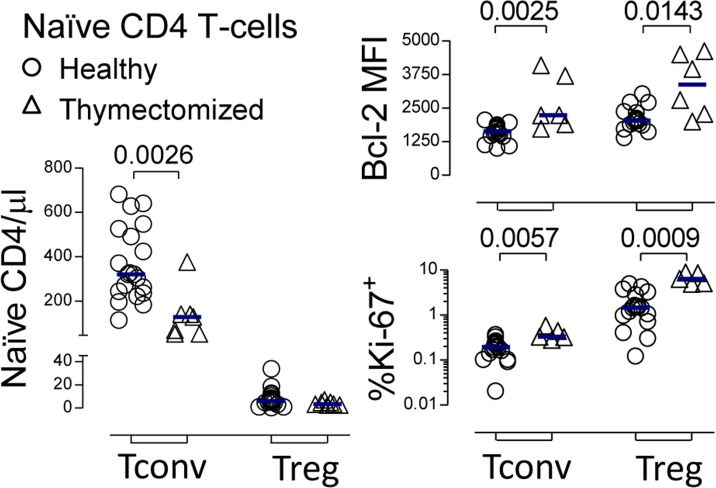
Preservation of the naïve-Treg compartment following thymus removal Circulating naïve-Treg and naïve-Tconv counts; as well as % of cycling-cells, and Bcl-2 MFI within these subsets in healthy and thymectomized adults; each symbol represents one individual; bars represent median; significant *P*-values of comparisons within each subset are shown.

We observed a significant decrease in circulating naïve-Tconvs, both in frequency within CD4 T-cells (*P* = 0.0042; Supplementary Figure 1A) and absolute numbers (*P* = 0.0026; Figure 1), in agreement with previous data from other thymectomized cohorts [11-14]. Conversely, the naïve-Treg pool size was preserved, as compared to healthy subjects (Figure 1 and Supplementary Figure 1A).

The healthy cohort spanned an age period associated with relatively stable thymic function and naïve-Treg numbers [6], and the size of the circulating naïve-Treg pool was within the range previously described [4-6]. We sorted naïve-Tregs, and confirmed that their *FOXP3* mRNA expression levels were comparable to those found in memory-Tregs (4526-21104 *versus* 1540-14363 relative copy numbers, respectively, *n* = 3 healthy adults), and much higher than those in naïve-Tconvs (34-115 relative copy numbers), confirming them as *bona fide* Tregs [1].

Circulating naïve-Tregs were confirmed to have a truly naïve phenotype both in healthy and thymectomized subjects, based on the expression of a panel of naïve markers and reduced CD95 expression, as well as to express Treg function-associated markers (CTLA-4, HLA-DR, CD39) at lower levels than memory-Tregs (Figure 1 and Supplementary Figure 1B). Although Helios has been proposed as a marker of thymus-derived Tregs, we showed that in both cohorts a significant proportion of circulating naïve-Tregs lacked Helios expression (Figure 1 and Supplementary Figure 1B), as already observed in human mature FoxP3^+^ CD4 single-positive (CD4SP) thymocytes (Supplementary Figure 1C), questioning its usefulness as a marker of thymic-derived Tregs [25]. Regarding the CD31^+^ subset, a population known to be enriched in recent thymic emigrants [16], no significant contraction was observed within naïve-Tregs of thymectomized, as compared to healthy individuals (*P* = 0.1708, Supplementary Figure 1B), in contrast to the significant reduction observed within naïve-Tconvs (*P* = 0.0122, Supplementary Figure 1B). This finding suggests that distinct homeostatic mechanisms are operating in the two naïve compartments upon thymectomy. Interestingly, we have previously shown that IL-7 up-regulates CD31 expression [17], further validating the importance of investigating the impact of IL-7 on naïve-Tregs, as detailed below.

In order to investigate the mechanisms underlying the preservation of the naïve-Treg compartment, we quantified the expression levels of markers of cell survival and cell cycling in thymectomized, as compared to healthy individuals. Bcl-2 expression was significantly higher, supporting a contribution of increased naïve-Treg survival to their maintenance upon thymectomy (*P* = 0.0143; Figure 1). Additionally, significantly higher frequencies of cycling-cells, as assessed by Ki-67, were documented (*P* = 0.0009; Figure 1). The degree of Bcl-2 increase in thymectomized was comparable in the two naïve compartments, whereas the increase in Ki-67^+^ cell frequency was much more striking within naïve-Tregs (4-fold) than within naïve-Tconvs (1.7-fold), supporting an important contribution of increased cell-turnover to the better preservation of the naïve-Treg compartment.

Altogether, our data indicate that naïve-Tregs are preserved in adults submitted to total thymectomy in early infancy, mainly through increased homeostatic proliferation.

### High steady-state turnover of the naïve-Treg compartment

The finding of a preserved naïve-Treg compartment in thymectomized adults prompted us to further investigate the mechanisms underlying naïve-Treg homeostasis under steady-state conditions in healthy young adults with stable thymic output.

**Figure 2:**
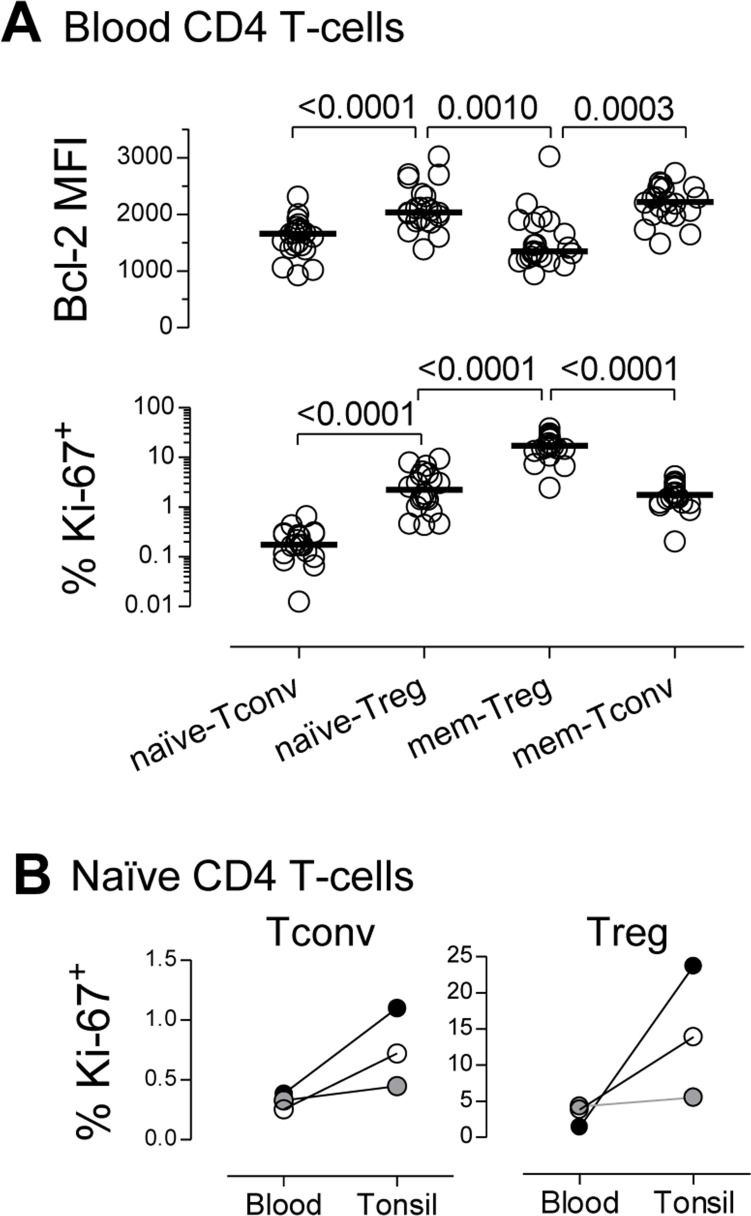
High steady-state turnover of the naïve-Treg compartment **A.** Bcl-2 MFI and % of cycling-cells, within circulating CD4 T-cell subsets; each dot represents one individual; bars represent median; significant *P*-values are shown. **B.** % of cycling-cells within naïve-Tregs and naïve-Tconvs from matched blood and tonsil samples (each symbol represents one child: 2years/open, 6years/grey, and 7years/black).

We found that naïve-Tregs displayed significantly higher levels of Bcl-2 than both naïve-Tconvs and memory-Tregs (Figure 2A), suggesting that enhanced survival plays a major role in their maintenance. Therefore, in this respect, naïve-Tregs are distinct from memory-Tregs, which have been shown to have a pro-apoptotic profile [1, 4].

Additionally, naïve-Tregs featured a much higher *in-vivo* turnover than naïve-Tconvs (Figure 2A), with significantly elevated proportion of cycling-cells (2.23%[1.12-4.75] *versus* 0.17%[0.10-0.29], *P* < 0.0001). Thus, although naïve-Tregs are frequently labeled as “quiescent” as opposed to memory-Tregs, we show here that they featured levels of cycling comparable to those found in memory-Tconvs (Figure 2A).

Homeostatic naïve-Tconv proliferation occurs mainly in SLO [16]. We performed a parallel study of blood and tonsils, used here as an example of SLO due to ease of access to clinical samples. Tonsil naïve-Tregs featured a much higher proportion of cycling-cells than blood naïve-Tregs (Figure 2B). Notably, the fold-increase in cycling-cells in tonsils as compared to blood was much more striking in the naïve-Treg than in the naïve-Tconv compartment (4-fold *versus* 2-fold, respectively). The presence of naïve-Tregs expressing Ki-67 in tonsil tissue was further confirmed by immunofluorescence (Supplementary Figure 2A). Additionally, we showed that naïve-Tregs from freshly-collected tonsils consistently entered S-phase at higher rates than naïve-Tconvs, as estimated by the *ex-vivo* incorporation of 5-ethynyl-2′-deoxy-uridine (EdU), a thymidine analogue (Supplementary Figure 2B; 12 hours incubation: naïve-Treg: 1.23-1.70%; naïve-Tconv: 0.37-0.42%; *n* = 3). Of note, in tonsils, naïve-Tregs featured significantly higher proportion of Ki-67^+^ cells than naïve-Tconvs (Figure 2B), as well as Bcl-2 MFI (mean fluorescence intensity; range 2777-3161 *versus* 1873-1992, *n* = 3, respectively). These results suggest that SLO may provide appropriate niches for naïve-Treg homeostatic proliferation.

Overall, our data show that, in healthy young adults, naïve-Tregs featured markers of enhanced survival and increased turnover in comparison to naïve-Tconvs, supporting an important contribution of peripheral proliferation to the maintenance of the naïve-Treg compartment.

### *Ex-vivo* evidence for ongoing naïve-Treg response to IL-7

**Figure 3:**
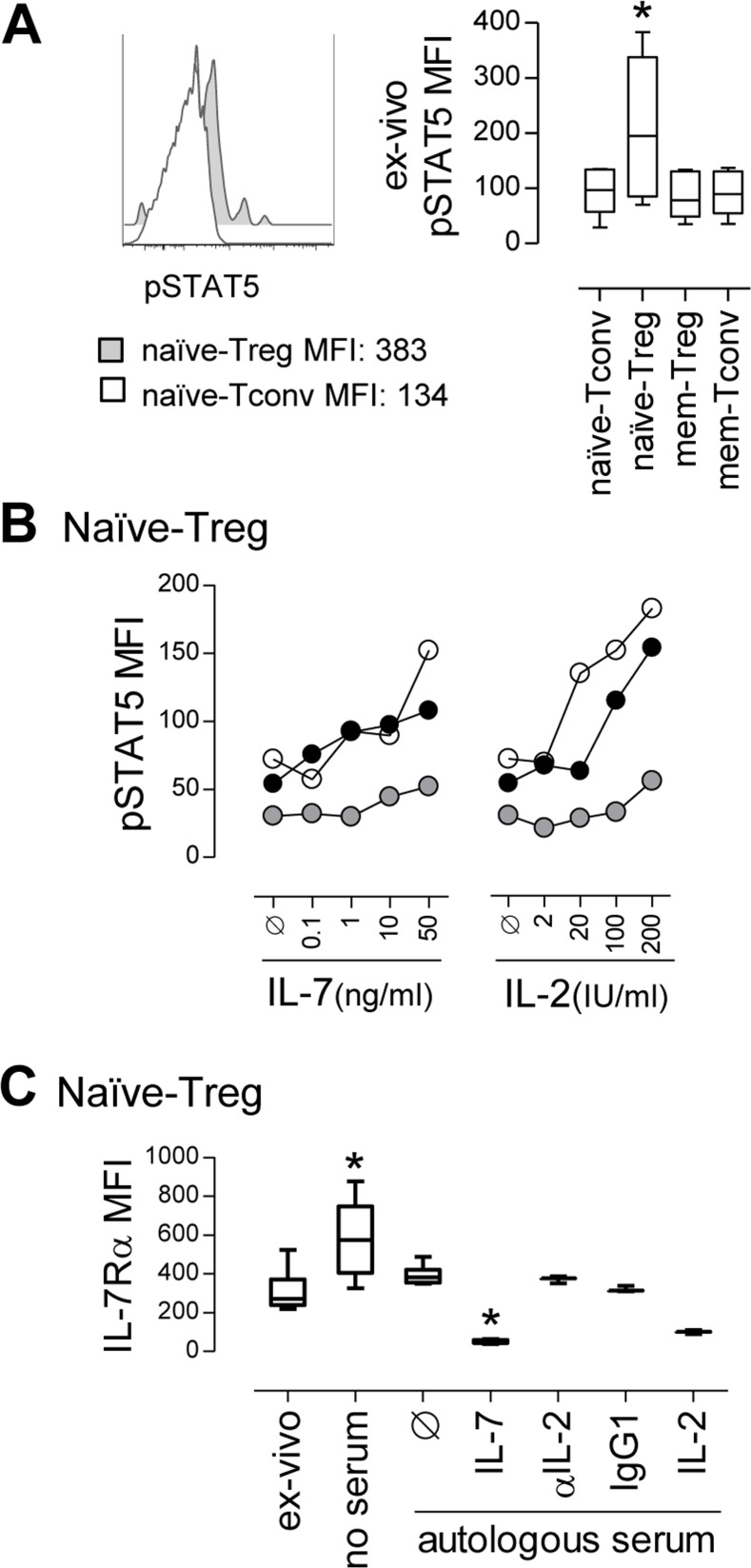
*Ex-vivo* evidence for ongoing naïve-Treg response to IL-7 **A.** Representative histogram of *ex-vivo* pSTAT5 levels in circulating naïve-Tregs and naïve-Tconvs, with graph showing pSTAT5 MFI within these and counterpart memory CD4 T-cell subsets; comparison of pSTAT5 levels only revealed significant differences between naïve-Tregs and all the other subsets (* *P* < 0.05; *n* = 6). **B.** Analysis of pSTAT5 MFI within gated naïve-Tregs upon *in-vitro* stimulation with increasing concentrations of IL-7 or IL-2; each symbol represents one individual. **C.** IL-7Rα MFI within naïve-Tregs analyzed *ex-vivo* and after 24 hour-culture without serum or supplemented with 40% autologous serum alone or with IL-7 (*n* = 6); as well as with IL-2, anti-IL-2 blocking monoclonal antibody, or isotype-control (*n* = 3); * *P* < 0.05 as compared to *ex-vivo* levels.

Next, we investigated the putative contribution of γc-cytokines to the increased turnover and survival of naïve-Tregs. *Ex-vivo* levels of pSTAT5, a downstream marker of both IL-7 and IL-2-mediated signaling, were significantly higher in naïve-Treg than in naïve-Tconv and memory CD4 subsets, analyzed in freshly-collected whole blood from healthy subjects (Figure 3A), irrespectively of the levels of expression of IL-7Rα and IL-2Rα (Supplementary Figure 1B). Additionally, we stimulated purified CD4 T-cells with increasing concentrations of IL-7 or IL-2, and clearly showed that naïve-Tregs responded to both cytokines, in a dose-dependent manner (Figure 3B and Supplementary Figure 3A). Our data extend previous human and murine studies on the ability of Tregs to phosphorylate STAT5 in response to IL-7 *in-vitro* [20, 26-28], through the comparison of naïve and memory Tregs, as well as their FoxP3 negative counterparts.

Since IL-7 signaling induces the down-regulation of its receptor [17], we reasoned that the level of recovery of IL-7Rα expression upon IL-7 deprivation would reflect the extent of ongoing IL-7 signaling *in-vivo*. IL-7Rα expression levels were quantified in purified CD4 T-cells from healthy subjects following 24 hours in different culture conditions (Figure 3C and Supplementary Figure 3B). Naïve-Tregs featured a significant recovery of IL-7Rα expression in the absence of IL-7 (culture in serum free medium), as compared to *ex-vivo* levels, in agreement with a significant ongoing IL-7 response. Notably, IL-7Rα expression was relatively preserved in the presence of autologous serum (IL-7 levels range: 15.8-23.5pg/ml). Moreover, the possible contribution of IL-2, either derived from serum or produced by cells in culture, was excluded by documenting a lack of impact of IL-2 blockade. Addition of exogenous IL-7 (10ng/ml) or IL-2 (20IU/ml) led to the expected down-regulation of IL-7Rα [29].

In conclusion, we showed that naïve-Tregs featured high *ex-vivo* pSTAT5 levels, and recovered IL-7Rα expression after IL-7 deprivation, supporting their ongoing response to IL-7 *in-vivo*.

### Preserved naïve phenotype, Treg-markers and suppressive capacity upon naïve-Treg response to IL-7

**Figure 4:**
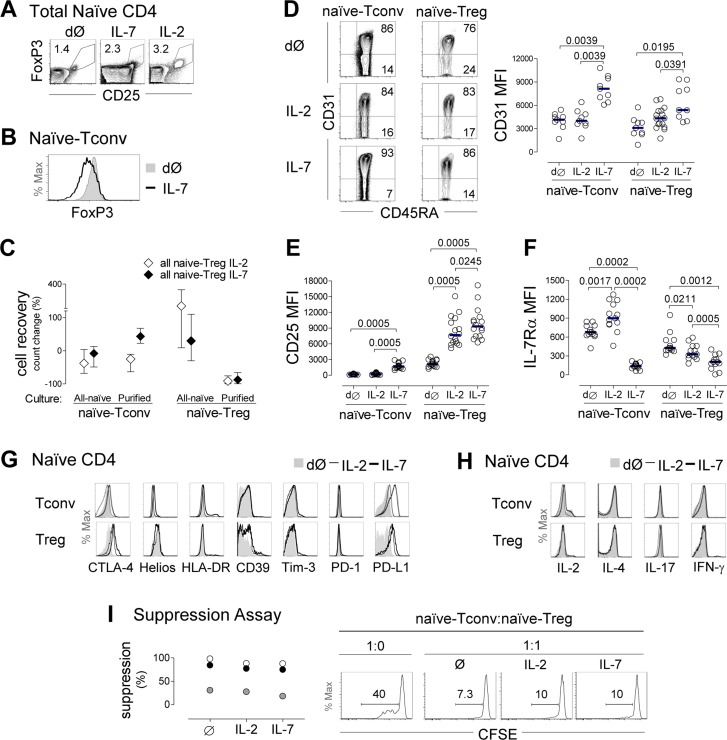
Evidence of naïve-Treg response to IL-7 while preserving their naïve and suppressive phenotype **A.** Representative contour-plots of FoxP3 and CD25 expression within total naïve CD4 T-cells *ex-vivo* (dØ) and upon 13 day-culture with IL-7 or IL-2. **B.** Illustrative histogram demonstrating absence of FoxP3 induction in purified naïve-Tconvs following 13 day-culture with IL-7. **C.** Cell recovery upon 13 day-culture with IL-7 or IL-2 of total naïve CD4 T-cells (*n* = 11), purified naïve-Tregs (*n* = 3) or purified naïve-Tconvs (*n* = 3); graph shows median and interquartile range or range (purified populations). **E.**-**F.** Analysis of naïve-Tregs and naïve-Tconvs *ex-vivo* and post-13 day-culture of naïve CD4 T-cells with IL-7 or IL-2 showing representative contour-plots of CD31/CD45RA expression (D), and graphs of MFI of CD31 (D), CD25 (E), and IL-7Rα (F); each dot represents one individual; bars represent median; comparisons done within each subset; significant *P*-values are shown. **G.** Illustrative histograms of CTLA-4, Helios, HLA-DR, CD39, Tim-3, PD-1 and PD-L1 expression within naïve-Tregs and naïve-Tconvs *ex-vivo* and post-13 day-culture with IL-7 or IL-2 (one/3-14). **H.** IL-2, IL-4, IL-17 and IFN-γ production after short-term PMA/Ionomycin stimulation *ex-vivo* and post-13 day-culture with IL-7 or IL-2 (one/3). **I.** Illustrative histograms of CFSE expression within naïve-Tconvs cultured alone or with naïve-Tregs pre-incubated with medium alone, IL-7 or IL-2 (numbers represent % of cells that divided at least once); graph shows % of suppression of naïve-Tconv proliferation in 3 individuals.

We subsequently investigated the impact of IL-7 on naïve-Treg phenotype and suppressive capacity. For this purpose, total naïve CD4 T-cells were purified from freshly-collected blood of healthy donors, and cultured for up to 13 days in the presence of IL-7 or IL-2, using conditions previously optimized in our laboratory [17]. Analysis was performed within naïve-Tregs and naïve-Tconvs defined according to their FoxP3 expression, as illustrated in Figure 4A.

We confirmed that FoxP3 was not induced in naïve-Tconvs in the presence of IL-7, using sorted naïve-Tconvs cultured alone with IL-7, and showing no increase in FoxP3 expression either at the protein level, by flow-cytometry (Figure 4B), or at the transcriptional level by real-time PCR (*ex-vivo* range 34-115 *versus* 14-62 FOXP3 relative copy numbers after 13 day-culture, *n* = 3). FoxP3 induction was also not observed in cultures of purified naïve-Tconvs with IL-2 (data not shown). This is in agreement with the requirement for TCR stimulation for *de novo* induction of FoxP3 in conventional CD4 T-cells [1].

Importantly, despite the clear impact of both IL-7 and IL-2 on the recovery of naïve-Tregs in cultures of total naïve CD4 T-cells, neither cytokine was able to maintain sort-purified naïve-Tregs when they were cultured alone (Figure 4C). This finding revealed a requirement for naïve-Tregs to interact with naïve-Tconvs to ensure their survival, which was not overcome by the addition of IL-2 (Figure 4C). These data precluded purified naïve-Treg culture in isolation.

Of note, the overall proportion of naïve-Tregs within total naïve CD4 T-cells significantly increased upon 13 day-culture with IL-7 (from 0.99%[0.60-1.43] to 2.15%[1.29-3.21], *P =* 0.0048, *n* = 16). This increase was more evident upon culture with IL-2 (7.94%[3.65-11.75], *P =* 0.0005), likely due to the reduced impact of IL-2 on naïve-Tconvs (Figure 4C).

As illustrated in Figure 4D, FoxP3^+^ cells maintained their naïve phenotype following 13 day-culture with either IL-7 or IL-2. Our previous work showed that IL-7 increases CD31 expression in naïve CD31^+^ CD4 T-cells [17]. Here, we showed that naïve-Tregs cultured in the presence of IL-7 up-regulated CD31 MFI to levels comparable to naïve-Tconvs (fold-increase: 2.17[1.45-3.69] *versus* 2.17[1.73-2.46], *P =* 0.4333, respectively; Figure 4D). Moreover, naïve-Tregs up-regulated CD25 (fold-increase: 3.85[3.296.01], Figure 4E), and down-regulated IL-7Rα (fold-reduction: 0.43[0.23-0.67], Figure 4F) upon IL-7 stimulation, although less markedly than naïve-Tconvs (11.54[7.80-14.96], *P* < 0.0001 for CD25; and 0.21[0.15-0.27], *P =* 0.0058 for IL-7Rα expression). These findings support an ability of naïve-Tregs to respond to IL-7.

The impact of IL-2 on naïve-Tregs followed a similar pattern, albeit significantly less striking than that observed for IL-7 (Figure 4D-4F). Conversely, in naïve-Tconvs both CD25 and CD31 expression levels were unaltered in response to IL-2, whereas IL-7Rα expression increased (Figure 4D-4F), possibly due to a dominant effect of IL-7 deprivation [17, 29].

Concerning Treg function-associated markers, naïve-Treg FoxP3 MFI (*ex-vivo*: 778[650-1009], *n* = 16) significantly increased upon culture with IL-7 (1166[863-1522], *P =* 0.0214), and even more so with IL-2 (1229[902-1916], *P =* 0.0280 as compared to *ex-vivo*, *P =* 0.0362 as compared to IL-7). Furthermore, we observed a sustained or moderate increase in CTLA-4, CD39 and HLA-DR expression upon culture with either IL-7 or IL-2 (Figure 4G), suggesting maintenance of regulatory function. Additionally, IL-7 induced a major up-regulation of PD-L1, but not PD-1, on both naïve subsets (Figure 4G).

Importantly, naïve-Tregs remained unable to produce IL-2, IL-4, IL-17 or IFN-γ after 13 day-culture in the presence of IL-7 or IL-2, as evaluated upon further short-term stimulation with PMA plus Ionomycin (Figure 4H).

Finally, we asked whether exposure to IL-7 impairs the suppressive capacity of naïve-Tregs. For this purpose we sorted naïve-Tregs and compared their suppressive capacity upon 4 hours pre-incubation with culture medium alone or supplemented with IL-7 or IL-2. We observed that upon treatment with either cytokine, naïve-Tregs maintained their ability to suppress autologous naïve-Tconv proliferation in response to TCR stimulation (Figure 4I).

Altogether, these results support an ability of naïve-Tregs to respond to IL-7 whilst maintaining both their naïve and suppressive phenotype.

### IL-7 induced naïve-Treg survival and proliferation

**Figure 5:**
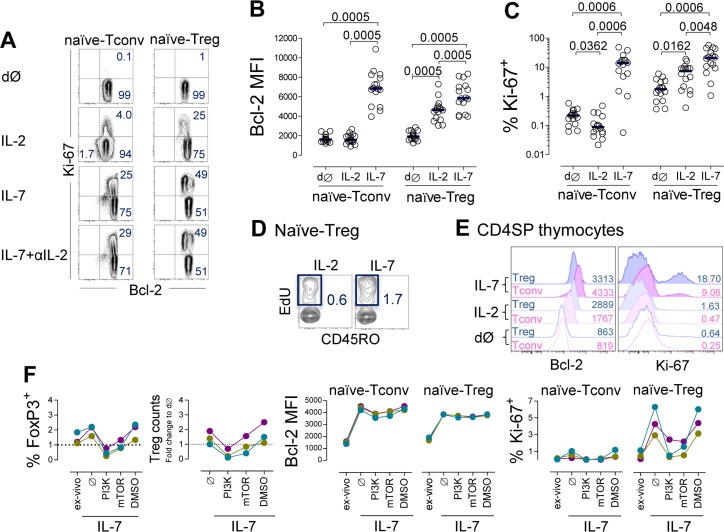
IL-7 induced naïve-Treg survival and proliferation **A.**-**D.** Analysis of naïve-Tregs and naïve-Tconvs *ex-vivo* (dØ) and post 13 day-culture of purified naïve CD4 T-cells with IL-7 or IL-2 or IL-7 plus anti-IL-2 blocking monoclonal antibody: (A) Representative contour-plots of Bcl-2 and Ki-67 expression; graphs show Bcl-2 MFI (B) and % of cycling-cells (C), bars represent median; significant *P*-values of comparisons within each subset are shown; (D) Contour-plots illustrating the impact of IL-7 or IL-2 on EdU incorporation by naïve-Tregs (final 12 hours-culture, *n* = 2). **E.** Illustrative histograms of Bcl-2 and Ki-67 expression within gated FoxP3^+^ or FoxP3^─^ cells *ex-vivo* and post 7 day-culture of purified CD4SP thymocytes with IL-7 or IL-2 (one/3). **F.** Impact of the PI3K-inhibitor (LY294002), mTOR-inhibitor (rapamycin), or vehicle control (DMSO), on naïve CD4 T-cells upon 7 day-culture with IL-7; graphs show % of naïve-Treg and change in cell recovery, Bcl-2 MFI and % of cycling-cells within naïve-Tregs and naïve-Tconvs, as compared to *ex-vivo* levels; each color represents one individual.

We then asked whether IL-7 induces the expression of markers of survival (Bcl-2) and cell cycling (Ki-67) in naïve-Tregs. We observed a clear induction of Bcl-2 expression in naïve-Tregs cultured in the presence of IL-7 (Figure 5A-5B), although less marked than in naïve-Tconvs (fold-increase: 3.02[2.33-3.72] *versus* 3.93[3.29-5.05], respectively, *P <* 0.0001). Bcl-2 up-regulation in naïve-Tregs was significantly higher upon IL-7 stimulation than with IL-2 (Figure 5A-5B), using optimal concentrations of both cytokines [17].

Additionally, IL-7 induced cycling of naïve-Tregs (Figure 5A and 5C). The proportion of cells expressing Ki-67 (21.06%[9.62-40.40]) was higher than that observed within naïve-Tconvs (13.92%[5.11-17.63], *P =* 0.0186), and within naïve-Tregs cultured with IL-2 (7.31%[1.74-9.35], *P =* 0.0019). These findings are even more striking considering the much higher IL-7Rα expression levels on naïve-Tconvs (Supplementary Figure 1B). We further showed that IL-7 was superior to IL-2 in promoting naïve-Treg entry into cell-cycle, *via* assessment of the frequency of EdU-incorporating cells in the last 12 hours of the culture (Figure 5D). Proliferative studies using carboxyfluorescein succinimidyl ester (CFSE)-labeled cells were not performed, as we have previously demonstrated that this approach does not reproducibly reveal cytokine-mediated proliferation of naïve CD4 T-cells [17].

Importantly, as shown in Figure 5A, IL-7 induction of Bcl-2 and cell cycling was unaltered in the presence of an anti-IL-2 blocking monoclonal antibody, thus supporting a major role for IL-7 in the maintenance of naïve-Treg homeostasis independently of IL-2.

Human FoxP3^+^CD4SP thymocytes were already able to proliferate in culture, and to up-regulate Bcl-2 in response to IL-7 (Figure 5E), in agreement with our previous data showing an increase of pSTAT5 in FoxP3^+^ thymocytes upon stimulation with IL-7 [30].

Finally, we investigated whether the signaling pathways involved in IL-7 response differed between naïve-Tregs and naïve-Tconvs. IL-7 signaling uses both JAK3/STAT5 and PI3K/Akt pathways to promote T-cell survival and proliferation [17]. We have previously demonstrated that IL-7-mediated naïve CD4 T-cell proliferation, but not Bcl-2 up-regulation, critically depends upon PI3K activity [17]. On the other hand, Treg metabolism is known to rely on mammalian target of rapamycin (mTOR) [2]. To investigate the relative importance of these pathways, we added the cell permeable PI3K-specific inhibitor (LY294002) or mTOR inhibitor (rapamycin) to naïve CD4 T-cells cultured with IL-7 or IL-2. A 7 day-culture assay was selected to avoid possible deleterious impact of long-term exposure to the inhibitors, and was found to be sufficient to reveal Bcl-2 and Ki-67 induction albeit at lower levels than after 13 day-culture (Figure 5F). Inhibition of the PI3K pathway led to a major decrease in naïve-Treg recovery and proliferation upon stimulation with IL-7. Conversely, Bcl-2 expression was not significantly altered. Similar trends were observed upon mTOR inhibition, supporting its participation in the signaling cascade leading to proliferation. Moreover, the addition of these inhibitors to IL-2 stimulated cultures generated comparable profiles in naïve-Tregs, indicating that these pathways are utilized by both cytokines (data not shown).

Overall, our *in-vitro* data reveal that both IL-7 and IL-2 are able to promote proliferation of naïve-Tregs *via* activation of PI3K/mTOR signaling, and that IL-7 promotes the survival and proliferation of FoxP3^+^ mature thymocytes and circulating naïve-Tregs.

## DISCUSSION

Naïve-Tregs are thymus-committed regulatory T cells with a high degree of self-reactivity that continuously replenish the activated memory-Treg pool. Our finding of a preserved naïve-Treg compartment in adults submitted to total thymectomy in infancy support the relevance of peripheral mechanisms to ensure naïve-Treg homeostasis. We further showed that in steady-state conditions human naïve-Treg homeostasis occurs through a combination of high turnover rate and extended survival mediated by IL-7 and/or IL-2, based on the following evidence: 1) higher levels of the pro-survival molecule Bcl-2 and proportion of cycling-cells than naïve-Tconvs in both blood and tonsils; 2) the highest *ex-vivo* levels of pSTAT5 as compared to other naïve/memory CD4 T-cell subsets, which further increases, in a dose-dependent manner, following IL-7 or IL-2 stimulation; 3) recovery of IL-7Rα expression upon IL-7 deprivation, consistent with an ongoing *in-vivo* response to IL-7; 4) response to IL-7 and IL-2 in culture indicated by up-regulation of Bcl-2, and induction of PI3K/mTOR-mediated proliferation; and 5) maintenance of naïve and suppressive phenotype upon exposure to IL-7. Additionally, we showed that naïve-Treg precursors, namely FoxP3^+^CD4SP thymocytes, already proliferate and up-regulate Bcl-2 in response to IL-7 stimulation *in-vitro*.

Thus, our results challenge the assumption that naïve-Tregs are a quiescent population in steady-state conditions in non-lymphopenic individuals [1]. Human naïve-Tregs have been shown to feature a functional and molecular profile partially overlapping with both memory-Tregs and naïve-Tconvs [1, 4, 6]. Regarding their homeostasis, as for the memory compartment we found a much higher cell-turnover in naïve-Tregs than in naïve-Tconvs, suggesting an intrinsic propensity of Tregs to enter cell cycle. On the other hand, in agreement with their naïve character, naïve-Treg turnover was lower and Bcl-2 levels higher than in their memory counterparts. Additionally, similar pathways were apparently operating in response to IL-7 in naïve-Tregs and naïve-Tconvs.

In fact, we revealed here, for the first time, a role for IL-7 in naïve-Treg survival and homeostatic proliferation. These findings are particularly relevant for understanding the impact of *IL-7/IL-7Rα* polymorphisms on autoimmunity [31], and the mechanisms underlying Treg expansion in lymphopenic settings, with implications for the therapeutic use of IL-7.

Our data do not exclude the contribution of self-peptide/MHC stimulation to the naïve-Treg proliferation, which may be particularly important given their enrichment in self-antigen reactivities [6]. However, TCR stimulation of naïve-Tregs has been associated with rapid acquisition of a memory phenotype, with the potential risk of contraction of the naïve compartment [1, 4]. Of note, the maintenance of murine naïve-like FoxP3^+^ CD4 T-cells was recently demonstrated to be independent of TCR-driven signals [32].

Importantly, we showed that naïve-Treg proliferation in response to IL-7 or IL-2 was associated with preservation of the naïve-like phenotype, in parallel with the maintenance or even increase of regulatory markers. We further showed that prior exposure of naïve-Tregs to IL-7 did not impair their suppressive capacity.

IL-7, like IL-2, is known to induce PD-1 and PD-L1 on Tconvs [33] and Tregs [20]. Our observations extended these reports, by showing that IL-7 up-regulated PD-L1, but not PD-1, on both naïve-Tregs and naïve-Tconvs. These results suggest that IL-7 contributes not only to T-cell homeostasis, but also to the containment of immunopathology, by up-regulating the inhibitory molecule PD-L1 on naïve CD4 T-cells, irrespective of their FoxP3 status. On the other hand, this may have implications for tumor immunity, given the clinically relevant suppression of tumor specific responses mediated by the PD-1/PD-L1 pathway [34].

SLO are an important source of IL-7 [10, 17]. Our results from tonsil tissue, using both flow-cytometry and immunofluorescence, confirmed a high local naïve-Treg turnover, suggesting that SLO contain appropriate niches for naïve-Treg homeostasis. Nevertheless, more detailed studies are required to decipher the topography of naïve-Tregs in SLO, as well as the contribution of their possible interactions with IL-7-producing stromal reticular cells, dendritic cells or IL-2-producing memory T-cells.

Our data argue for the sufficiency of peripheral proliferation and survival to achieve long-term maintenance of the naïve-Treg compartment even in the absence of thymic output. These results in adults expand upon a recent report describing preservation of naïve-Treg counts during short-term follow-up of children submitted to total thymectomy in early infancy [15]. Although a previous study showed a reduction of naïve-Treg frequency within total Tregs [13], this was not related to an actual contraction of naïve-Tregs upon thymectomy, but rather to the relatively high frequency of total Tregs, as also observed in our study (Table 1; proportion of naïve-Tregs within total Treg compartment: 11.60%[9.08-18.00] in thymectomized *versus* 14.85%[12.05-23.95] in healthy subjects, *P* = 0.3776).

The increase in naïve-Treg cycling in thymectomized adults was more likely mediated by IL-7 than by self-peptide/MHC interaction, given the conserved frequency of CD31^+^ cells, which are typically lost following TCR stimulation [16], and maintained by IL-7 [17]. We further showed that the CD31^+^ subset of naïve-Tregs was preferentially expanded by IL-7 *in-vitro*, in agreement with our previous data generated with total naïve CD4 T-cells [17]. Adults thymectomized in infancy feature an unexpectedly low prevalence of autoimmune diseases and allergy [12], which may be related to the maintenance of the broadly-reactive naïve-Treg pool.

To our knowledge, there are no published data specifically addressing the changes in the naïve-Treg compartment in individuals receiving IL-7 therapy. Nevertheless, an increase in total Treg numbers was documented in clinical trials, although the expansion of the Tconv compartment was much higher, leading to maintenance [35, 36] or a relative decrease [37-39] in Treg proportions. In agreement with our *in-vitro* data, naïve-Treg expansion was reported upon IL-2 therapy [28, 40, 41].

In severe lymphopenic settings associated with elevated serum IL-7 levels, such as HIV infection [42], there is an apparent preservation of circulating naïve-Tregs [43], raising the possibility that these cells are responding to IL-7. We did not find a correlation between the size of the naïve-Treg compartment and IL-7 serum levels in either our thymectomized individuals or the study population as a whole, which may be due to the narrow range of IL-7 levels (Table 1). Our data are also relevant in other lymphopenic settings, particularly in the context of immune reconstitution following hematopoietic stem cell transplantation, where IL-7-driven proliferation of naïve-Tregs may help to control graft *versus* host disease. Our results support a model in which the increased availability of IL-7 in lymphopenic settings may contribute to the expansion of the naïve-Treg compartment, thereby helping prevent immunopathology in the context of a constrained TCR repertoire.

In conclusion, naïve-Tregs feature high turnover in healthy adults, which further increases to compensate the loss of thymic replenishment upon total thymectomy. Moreover, our data reveal a role for IL-7 in naïve-Treg maintenance, both through up-regulation of the survival molecule Bcl-2 and induction of PI3K/mTOR-mediated proliferation. Clinical use and evaluation of therapies targeting the IL-7 pathway should take into account the contribution of IL-7 to naïve-Treg homeostasis.

## MATERIALS AND METHODS

### Study design

Blood was collected from healthy and age-matched adults thymectomized in infancy (Table 1). Complete thymectomy was confirmed based on the corrective cardiac surgery report and severely reduced levels of sjTREC. Patients with syndromatic cardiopathy were excluded. Circulating naïve-Tregs were further compared to their precursors, CD4SP thymocytes, isolated from thymic tissue obtained from children during routine corrective cardiac surgery. Tonsil tissue and blood collected at the same time from children submitted to tonsillectomy were used to study in parallel naïve CD4 T-cells in blood and SLO. All subjects/legal guardians gave written informed consent for blood and/or tissue sampling. Study was approved by Ethical Boards of Faculty of Medicine of University of Lisbon, and from Hospital de Santa Cruz for thymic tissue collection, Portugal.

### Cell isolation

PBMCs were isolated from freshly-collected heparinized blood *via* Ficoll-Paque PLUS (GE Healthcare, Uppsala, Sweden). Total or naïve CD4 T-cells were subsequently purified by negative selection (purity >96%, StemCell Technologies, Grenoble, France). Naïve-Tregs (CD45RO^─^CD25^high^CD127^─^), naïve-Tconvs (CD45RO^─^CD25^─^CD127^high^), and memory-Tregs (CD45RO^+^CD25^high^CD127^─^) were sorted from purified CD4 T-cells using a FACSAria (purity >95%; BD Biosciences, San Jose, CA). Tonsillar mononuclear cells and thymocytes were recovered using Ficoll-Paque PLUS after mechanical dispersion of tonsil and thymic tissue. CD4SP thymocytes were subsequently purified as CD3^high^CD8^─^CD4^+^ cells using a FACSAria (purity >97%).

### Cell culture

Purified naïve CD4 T-cells, naïve-Tregs or naïve-Tconvs, were cultured at 1×10^6^ cells/ml in complete medium as described [17], with either IL-7 (10ng/ml; R&D Systems, Minneapolis, MN) or IL-2 (20IU/ml; NIH/AIDS Research and Reference Program, Division of AIDS, NIAID, Hoffman-La Roche), for up to 13 days, with media replacement at day 3 and day 7. Naïve CD4 T-cell recovery per well was significantly higher upon culture with IL-7 than IL-2 (200,615[144,000-262,167] *versus* 95,958[60,063-178,125]; *n* = 16; *P* = 0.0006). In some experiments, anti-IL-2 blocking monoclonal antibody (clone 5334, 10μg/ml, R&D Systems) or isotype control IgG1 (10μg/ml, eBioscience, San Diego, CA) was added to cultures with IL-7. Cultures were also performed for 7 days in presence of PI3K inhibitor LY294002 (10μM, Merck Biosciences, Nottingham, UK); mTOR inhibitor rapamycin (100nM, Sigma-Aldrich); or drug vehicle DMSO (Sigma-Aldrich). For serum deprivation experiments, purified CD4 T-cells were cultured for 24 hours in medium without AB serum, or with 40% autologous serum alone or supplemented with IL-7, IL-2, anti-IL-2 blocking monoclonal antibody, or isotype control.

### Flow-cytometry

*Ex-vivo* phenotypic analysis was performed in freshly collected whole blood. Eight-color staining was performed using monoclonal antibodies listed in Supplementary Table 1. Intracellular staining was done using eBioscience FoxP3 kit. At least 150,000 events were acquired for each sample on a BD LSRFortessa (BD Biosciences) and data analyzed using FlowJo software (TreeStar, Ashland, OR). Dead cells were excluded according to FSC/SSC characteristics and/or LifeDead staining. After lymphogate definition, doublets were excluded, and cells analyzed within the mentioned gates.

### STAT5 phosphorylation

pSTAT5 was quantified in whole blood immediately after collection. After surface staining, red blood cells were lysed, and cells fixed and permeabilized using eBioscience FoxP3 protocol, followed by BD Cytofix and BD Phosflow (BD Biosciences), and 1 hour incubation at 4°C with anti-pSTAT5 monoclonal antibody and other intracellular markers (Supplementary Table 1). pSTAT5 was also quantified upon stimulation of surface stained purified CD4 T-cells, with increasing concentrations of recombinant IL-7 (0.1/1/10/50ng/ml) or IL-2 (2/20/100/200IU/ml) for 15 minutes at 37°C, as described [44].

### Thymidine analogue 5-ethynyl-2′-deoxy-uridine (EdU) incorporation

EdU (5μM) was added to cell cultures for 12 hours. Immediately after collection, cells were surface and intracellularly stained, resuspended in ice-cold 1% formaldehyde (15 minutes), kept on ice on 70% ethanol (10 minutes), and then washed several times in PBS with 0.05% Triton-X100 before detection of EdU-substituted DNA using the Click-iT^®^ EdU HCS Assay (Thermo Fischer Scientific) according to manufacturer's instructions.

### Cytokine quantification

Cytokine production at the single-cell level was assessed after 4 hours stimulation with PMA+Ionomycin and Brefeldin A, as described [44]. Serum IL-7 levels were quantified using Human IL-7 Quantikine HS ELISA kit (R&D Systems) [42].

### *In vitro* suppression assay

Purified naïve-Tregs were incubated with IL-7, IL-2, or medium alone for 4 hours followed by co-culture (ratio 1:1) with autologous naïve-Tconvs labeled with CFSE, as described [17], stimulated with plate bound anti-CD3 (0.5μg/ml, clone OKT3, eBiosciences) in the presence of irradiated autologous PBMCs (4000rad). CFSE intensity decline was assessed at day 4 by flow-cytometry; % of suppression of naïve-Tconv proliferation = [(% proliferating naïve-Tconvs plated alone - % proliferating naïve-Tconvs co-cultured with Treg)/% proliferating naïve-Tconvs plated alone]x100.

### sjTREC quantification

A highly sensitive nested quantitative PCR assay (detection-limit 1copy/10^5^ cells) was used as described [24]. Triplicate multiplex PCR amplification of sjTREC, together with the CD3γ chain was performed on lysed PBMCs; quantifications were then performed using a LightCycler™ in independent experiments, with the same first-round serial dilution standard curve.

### FOXP3 mRNA quantification

Total RNA (Zymo Research kits) was used to generate cDNA (Superscript III Reverse Transcriptase, Thermo Fischer Scientific). mRNA levels of *FOXP3* (primers/probe described in [45]), and *GAPDH* (Taqman Gene Expression Assay) were quantified in duplicates (ViiA 7 Real-Time PCR System, all from Thermo Fischer Scientific) using standard curves generated by serial dilutions of cDNA from pooled PBMCs for *GAPDH* and a plasmid with *FOXP3* sequence. Relative copy numbers of *FOXP3* were calculated upon normalization to *GAPDH*.

### Immunofluorescence staining

Human tonsils were placed in OCT (VWR, Radnor, PA) and snap-frozen in liquid nitrogen. 3μm sections were stained overnight at 4°C using antibodies listed in Supplementary Table 1, and DAPI as a nuclear counter stain. Image processing was performed using FIJI software.

### Statistical analysis

Statistical analysis was performed with Graph Prism Version 5.01 (GraphPad Software, San Diego, CA). The following tests were used: Friedman for variance; Wilcoxon-Signed Rank for pairwise comparisons; Mann-Whitney for unpaired comparisons; Spearman's coefficient for correlations. Results were expressed as median [interquartile range or range (*n* < 4)]. *P*-values < 0.05 were considered significant.

## SUPPLEMENTARY MATERIAL TABLE AND FIGURES


