# Supplementary material for: Human naïve regulatory T-cells feature high steady-state turnover and are maintained by IL-7
Source: Oncotarget. 2016 Feb 19;7(11):12163–75. doi: 10.18632/oncotarget.7512 (PMC4914276; doi:10.18632/oncotarget.7512)
Supplement: Supplementary file 1 [file oncotarget-07-12163-s001.pdf]

Human naïve regulatory T-cells feature high steady-state turnover and are maintained by IL-7

Supplementary Material

Supplementary Table 1. Antibodies used in Flow-Cytometry and Immunofluorescence

Antibodies for Flow Cytometry			
**Antibody**	**Fluorochrome**	**Clone**	**Source**
CD3	PerCP-Cy5.5/eFluor450	OKT3	eBioscience [a]
CD3	APC	UCHT1	eBioscience
CD3	V500	UCHT1	BD Bioscience [b]
CD4	PE/PerCP-Cy5.5/PE-Cy7/APC	RPA-T4	eBioscience
CD4	V500	RPA-T4	BD Bioscience
CD11c	FITC	3.9	eBioscience
CD14	FITC	61D3	eBioscience
CD19	FITC	HIB19	eBioscience
CD123	FITC	6H6	eBioscience
CD45RA	FITC/PerCP-Cy5.5/APC	HI100	eBioscience
CD45RA	eFluor450	2D1	eBioscience
CD45RA	PE-Cy7	L48	BD Bioscience
CD45RO	FITC/PerCP eFluor710/APC-H7	UCHL1	eBioscience
CCR7	FITC	150503	R&D Systems [c]
CCR7	PE	3D12	R&D Systems
CD25	PE-Cy7	2A3	BD Bioscience
CD27	PE/PE-Cy7	O323	eBioscience
CD31	PE/APC	WM-59	eBioscience
CD39	FITC/APC	eBioA1	eBioscience
CD95	PE-Cy7	DX2	eBioscience
CD127	Alexa Fluor660/APC eFluor780	eBioRDR5	eBioscience
HLA-DR	FITC	L243	BD Bioscience
HLA-DR	V500	G46.6	BD Bioscience
PD-1	FITC	MIH-4	eBioscience
PD-L1	APC	BIH1	eBioscience
FoxP3	PE/PerCP-Cy5.5/eFluor450	PCH101	eBioscience
CTLA-4	PE	BNI3	BD Bioscience
Helios	Alexa Fluor488	22F6	Biolegend [d]
Ki-67	FITC/PE/PerCP-Cy5.5/Alexa Fluor647	B56	BD Bioscience
Bcl-2	FITC	124	DAKO [e]
IL-2	Brilliant violet421	MQ1-17H12	Biolegend
IL-4	APC	8D4-8	eBioscience
IL-17A	PerCP-Cy5.5	eBio64DEC17	eBioscience
IFN-γ	PE-Cy7	4S.B3	eBioscience
pSTAT5	PerCP-Cy5.5	47/pY694	BD Bioscience
LIVE/DEAD®	APC-Cy7	-	Thermo Fischer Scientific [f]
**Primary Antibodies for Immunofluorescence**			
**Antigen**	**Species/isotype**	**Clone**	**Source**
FoxP3	Mouse/IgG1	236/E7	eBioscience
FoxP3	Rat/IgG2a	PCH101	eBioscience
Ki-67	Mouse/IgG1	B56	BD Bioscience
CD45RO	Mouse/IgG2a	UCHL1	eBioscience
CD3	Rabbit/NA	Polyclonal	Abcam [g]
**Secondary Antibodies for Immunofluorescence**			
**Specificity**	**Species**	**Fluorochrome**	**Source**
Mouse IgG1	Goat	Alexa Fluor 488	Thermo Fischer Scientific
Mouse IgG2a	Goat	Alexa Fluor 546	Thermo Fischer Scientific
Rabbit	Goat	Alexa Fluor 647	Thermo Fischer Scientific

Rat	Goat	Alexa Fluor 488	Thermo Fischer Scientific
Mouse IgG1	Goat	Alexa Fluor 555	Thermo Fischer Scientific
Mouse IgG2a	Goat	Alexa Fluor 633	Thermo Fischer Scientific

[a] eBioscience, San Diego, CA; [b] BD Bioscience, San Jose, CA; [c] R&D Systems, Minneapolis, MN; [d] Biolegend, San Diego, CA; [e] DAKO, Glostrup, Denmark; [f] Thermo Fischer Scientific, Walthan, MA; [g] Abcam, Cambridge, UK. NA- Not Applicable

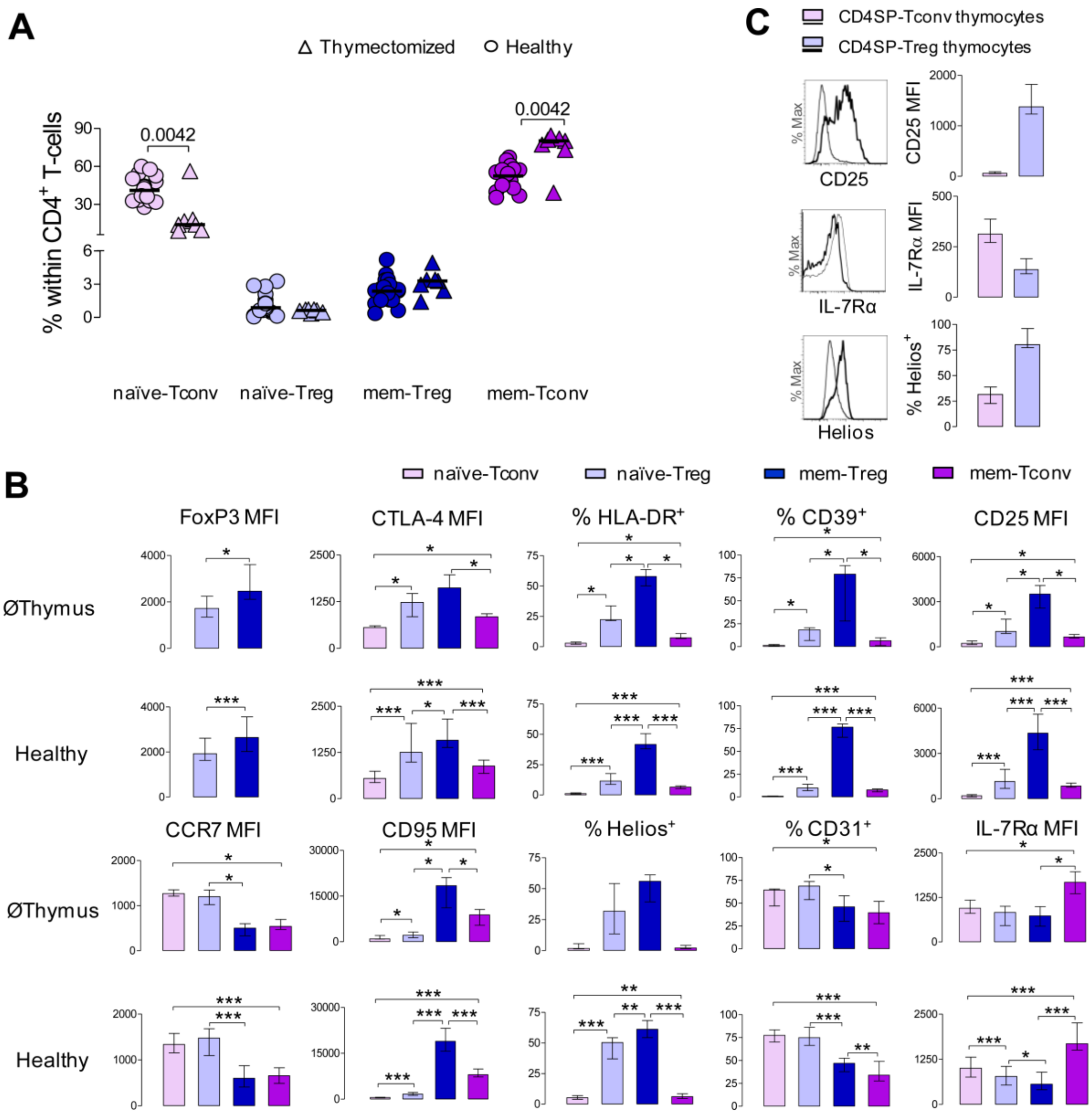

Supplementary Figure 1. ***Imbalances of CD4 T-cell subsets in adults submitted to total thymectomy in infancy.*** **(A)** Frequency of naïve-Tconv, naïve-Treg, mem-Treg, and mem-Tconv within circulating CD4$^+$ T-cells in thymectomized and age-matched healthy subjects; each dot represents one individual; comparisons performed for each subset between the two cohorts; significant *P*-values are shown. **(B)** Phenotype of the subsets **(**numbers of thymectomized and healthy, respectively, shown in brackets): FoxP3 (7/22), CTLA-4 (6/21), HLA-DR (7/22), CD39 (7/22), CD25 (6/21), CCR7 (7/21), CD95 (7/21), Helios (5/17), CD31 (7/16), and IL-7Rα (6/16); subsets within naïve and memory, as well as within Treg and Tconv compartments were compared; bars represent median and range; significant *P*-values are shown: * *P*<0.05, ** *P*<0.005, *** *P*<0.001. **(C)** CD25, IL-7Rα and Helios, within FoxP3$^+$ (CD4SP-Treg) and FoxP3$^-$ (CD4SP-Tconv) CD4SP thymocytes from three children (1, 3, and 9 months-old); bars represent median and range; % of mature CD4SP thymocytes (CD45RA^{bright}) lacking Helios expression varied between 4.5% and 19.6%.

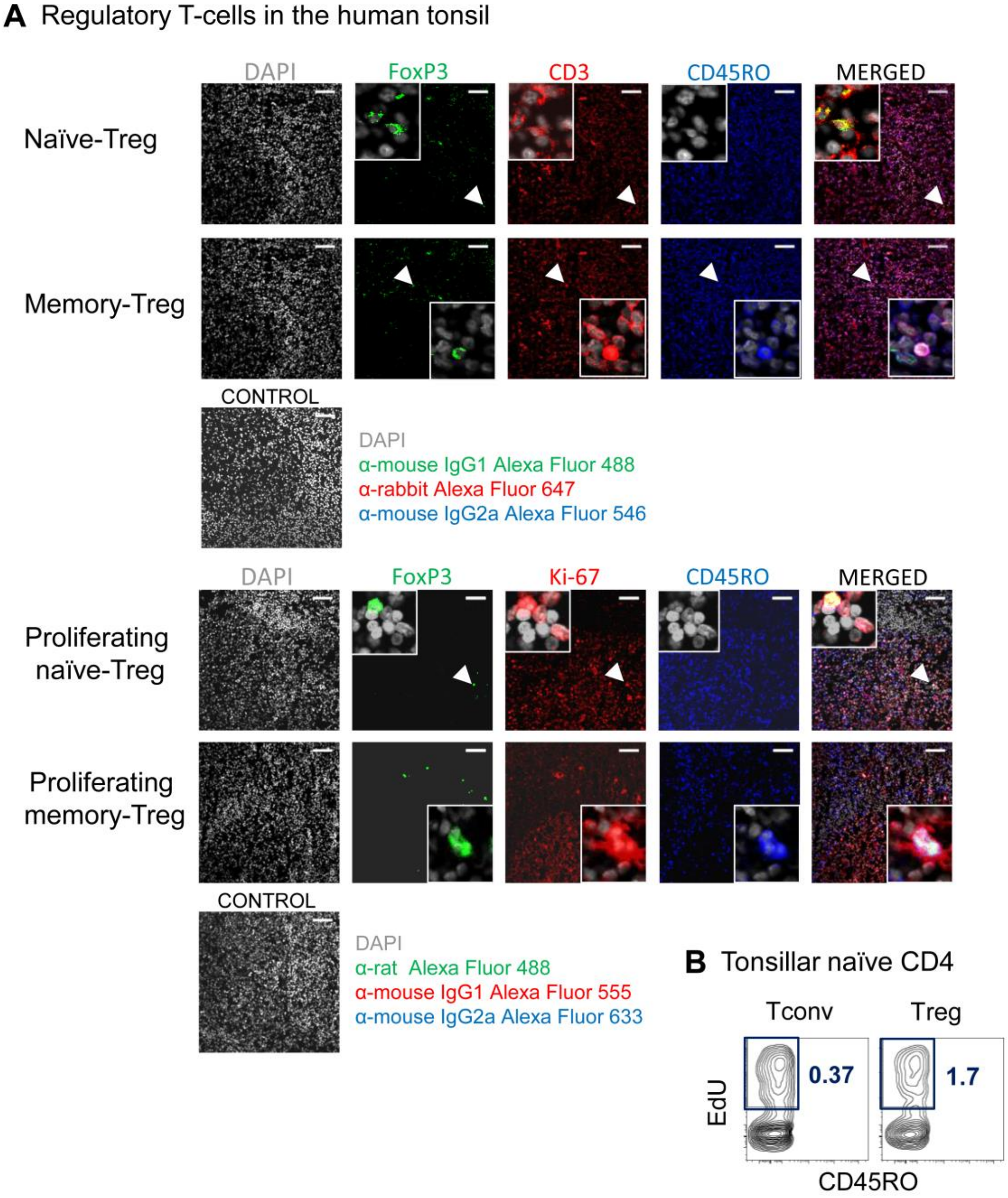

Supplementary Figure 2. ***Naïve-Treg proliferation in the human tonsil.*** **(A)** Illustrative immunofluorescence analysis of regulatory T-cells in human tonsil showing naïve ($CD3^{+}CD45RO^{-}$) and memory ($CD3^{+}CD45RO^{+}$) $FoxP3^{+}$ Treg in the top; and proliferating (Ki-67^{+}) naïve-Treg ($CD45RO^{-}FoxP3^{+}$) and memory-Treg ($CD45RO^{+}FoxP3^{+}$) in the bottom; DAPI was used to counter-stain nuclei. Illustrative images of single stainings, merged image, as well as the control staining in the absence of the primary antibodies are shown (resolution: 1024x1024; insert magnification: 200x, scale bar: 2.409 pixels/µm; length shown: 60µm); images were acquired using a Zeiss LSM710 microscope (Zeiss, Oberkochen, Germany) with a dry objective Plan-Apochromat with a numerical aperture of 0.8. **(B)** Contour-plots illustrating proportion of tonsillar naïve-Tregs and naïve-Tconvs incorporating EdU upon 12 hours-culture of freshly-isolated CD4 T-cells.

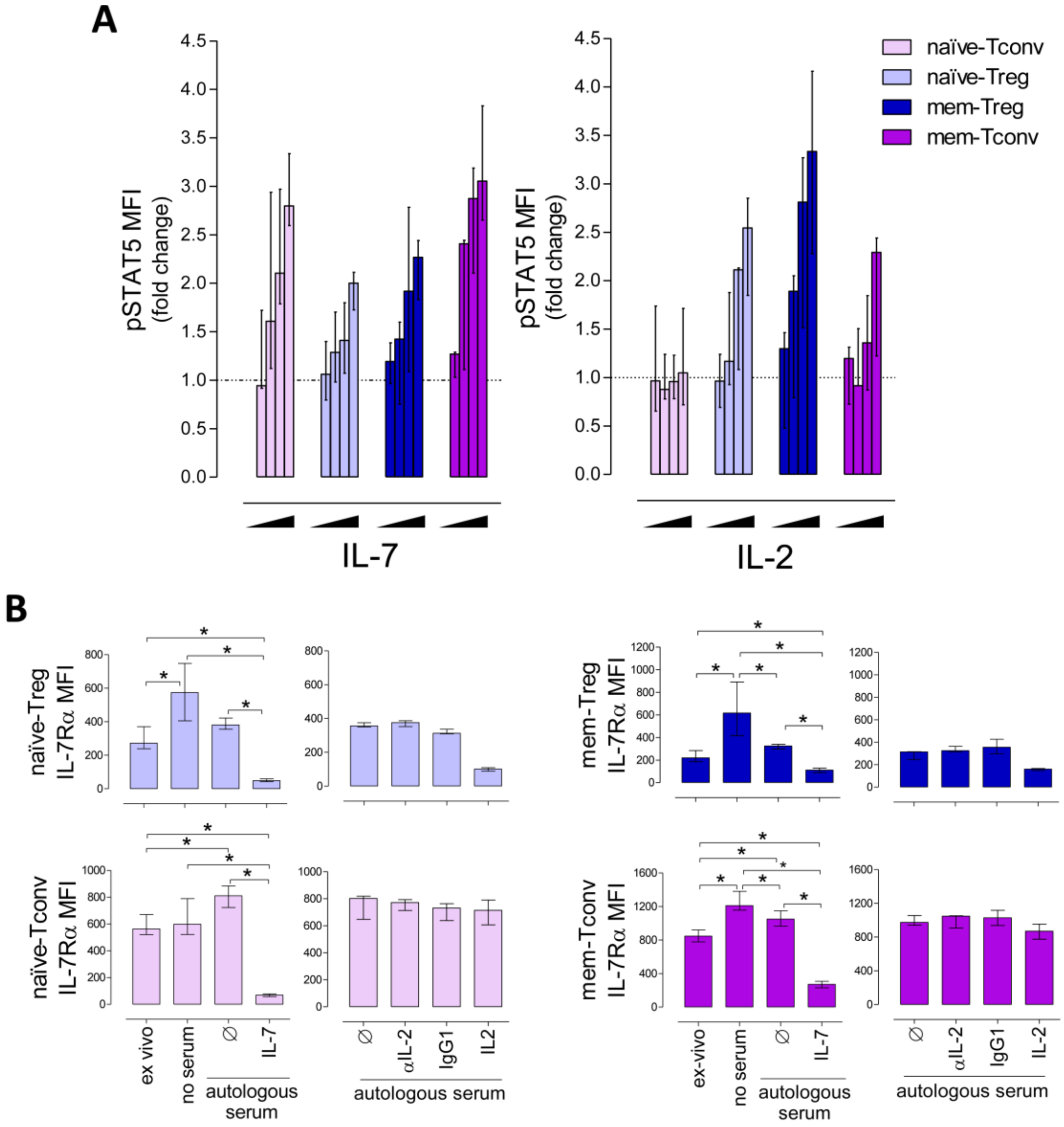

Supplementary Figure 3. ***Ex-vivo evidence of naïve-Treg response to IL-7.*** **(A)** IL-7 and IL-2 dose-dependent induction of pSTAT5 following 15 minutes *in-vitro* stimulation of purified CD4 T-cells; graphs show pSTAT5 MFI fold change in relation to non-stimulated cells in the gated CD4 T-cell subsets in three individuals; bars represent median with range. **(B)** IL-7Rα modulation in CD4 T-cell subsets according to exposure to IL-7 or IL-2; purified CD4 T-cells were cultured for 24 hours in the absence of IL-7/IL-2 (no serum), or with 40% autologous serum either alone or further supplemented with: IL-7 (10ng/ml), IL-2 (20IU/mL), anti-IL-2 blocking mAb (10μg/mL), or isotype-control (IgG1, 10μg/mL); graphs show the IL-7Rα MFI within gated CD4 T-cell subsets *ex-vivo* and post-culture, in six (left-hand graphs) and three (right-hand graphs) individuals; bars represent median with interquartile range (left-hand graphs; significant *P*-values are shown: *P<0.05) or median with range (right-hand graphs).
